# Progress of Obstetrics

**Published:** 1875-03

**Authors:** E. Warren Sawyer

**Affiliations:** Lecturer on Obstetrics, Rush Medical College, Chicago


ARTICLE II.

### PROGRESS OF OBSTETRICS.

BY E. WARREN SAWYER, M.D ,

LECTURER ON OBSTETRICS, RUSH MEDICAL COLLEGE, CHICAGO.

1. A Case of Immediate Transfusion. By JAMES R. CHADWICK, M.D. (*Boston Medical and Surgical Journal*, Jan. 14, 1875.)

1. The subject of the transfusion was a middle aged woman, who had suffered a profuse hæmorrhage in her second labor, which took place eleven weeks before her entrance into the hospital. In the meantime hæmorrhages had frequently recurred. Her health was undermined; she had frequent attacks of syncope, and persistent headache; her surface was completely blanched and the blood was greatly impoverished, as elicited from a microscopical comparison with healthy blood.

The usual measures had been invoked for a week in the hospital, despite which she was evidently sinking, and transfusion was employed as a last expedient. A loud souffle was heard with the heart's first sound; pulse 118; temperature 102.8 degrees.

The vein of the patient's arm was made to communicate with the vein of one of the medical gentlemen present, through Aveling's instrument. The operator intended to transfuse six ounces, but being wrongly informed of the capacity of the bulb of his instrument, he was made to transfuse eleven ounces—a mistake to which he attributes the fatal result.

During the operation, the pulse fell from 118 to 104. Immediately afterward there was some retching, but soon the patient expressed herself as better, and free from headache. Shortly after the operation, the patient had a chill which continued for an hour, after which the temperature rose to 104.6 degrees. There was frequent vomiting throughout the day. A troublesome hæmorrhage from the incised vein necessitated, finally, the tying of the vein, after the patient had lost four or five ounces of blood in the bed.

The woman failed gradually, and died on the evening of the same day.

The necropsy revealed some patches of a thin, translucent membrane, containing hæmorrhagic spots, upon the internal surface of the dura mater, marked anæmia of all the organs of the body, except the spleen ; also a fatty degeneration in spots on the endocardium and in the substance of the heart. There were no air bubbles or coagula of blood, as a result of the operation, though the entire vascular system was carefully searched for these.

The writer very properly says that the death of this woman was inevitable, though it was probably hastened by the strain to which the heart was subjected by increasing the volume of the circulation.

We would remark that it is further probable that death would have as surely followed if the operator had transfused but six ounces, as he intended, instead of eleven ounces ; in fact, it is questionable if the patient's veins retained even six ounces of the quantity transfused, for we are definitely informed that four or five ounces of blood were lost in the patient's bed, after the attempt was made to arrest the bleeding. Consequently we doubt that the mistake of the operator was an important factor in the cause of death.

Transfusion, of course, will not be resorted to, with any hope of success, in any chronic disease accompanied with marked organic change; on the contrary, there are some diseases that do most positively proscribe this expedient; foremost among them is fatty degeneration of the heart, or arteries. Under these circumstances, or in the course of pulmonary consumption, when the disease has weakened the heart, it is a hazardous thing to increase the volume of the blood ; and transfusion, if resorted to at all, should only be done while the impoverished blood of the patient is as rapidly escaping through the opened vein of the opposite arm ; an expedient early recommended, and which might have been invoked in this case, could fatty degeneration have been foretold.

There are no means of knowing positively the beginning of fatty degeneration. Dr. Chadwick has alluded to the experiments of Perl, who was able to develop fatty degeneration in the heart of dogs by bleeding them largely a few times. If then, a profuse hæmorrhage is a cause of fatty degeneration, is it not true that transfusion is strongly *contra-indicated in a case of hæmorrhage which occurred some weeks previously?*

The result in this instance detracts nothing from the value of transfusion employed at the moment of hæmorrhage.

In the above case, the supplying of a good quality of blood did not correct the souffle heard over the patient's heart. Considering this, and the fact that fatty degeneration of the heart was found *post mortem*, Dr. Chadwick asks if further investigation will not demonstrate that the souffle, instead of depending upon an impoverished condition of the blood, is really symptomatic of a fatty heart?

It is not our province to examine into this subject; still we cannot accept this connection in most instances of so-called inorganic murmurs, for we have known this souffle to disappear after a tonic treatment; too soon afterward to allow of the thought that a fatty degeneration of the heart's lining could have been recovered from, if, indeed, this condition is ever ameliorated.

